# The effect of early versus late initiation of renal replacement therapy in patients with acute kidney injury: A meta-analysis with trial sequential analysis of randomized controlled trials

**DOI:** 10.1371/journal.pone.0174158

**Published:** 2017-03-22

**Authors:** Yan-mei Feng, Yuan Yang, Xiao-li Han, Fan Zhang, Dong Wan, Rui Guo

**Affiliations:** 1 Department of Respiratory and Critical Care Medicine, The First Affiliated Hospital of Chongqing Medical University, Chongqing, P.R.China; 2 Department of Cardiovascular Medicine, The First Affiliated Hospital of Chongqing Medical University, Chongqing, P.R.China; 3 School of Public Health and Health Management, Chongqing Medical University, Chongqing, P.R.China; 4 Department of Critical Care Medicine, The First Affiliated Hospital of Chongqing Medical University, Chongqing, P.R.China; University of Sao Paulo Medical School, BRAZIL

## Abstract

**Background:**

The optimal timing for initiating renal replacement therapy (RRT) in patients with acute kidney injury (AKI) remains controversial.

**Methods:**

We conducted a meta-analysis with trial sequential analysis (TSA) of randomized controlled trials (RCTs) using PUBMED, Cochrane Library databases, and Web of Science (from January 1, 1985, to August 21, 2016). Adult patients with AKI who received RRT with different timing were included. The primary outcome was mortality. The secondary outcomes were intensive care unit (ICU) length of stay (LOS) and hospital LOS.

**Results:**

We included 9 RCTs with a total of 1636 participants. No differences between the early RRT group and the late RRT group were found with respect to mortality (38% vs 41.4%; relative risk, 0.93; 95% confidence interval [CI], 0.74–1.18). However, TSA showed that the cumulative Z-curve did not cross either the conventional boundary for benefit or the trial sequential monitoring boundary, indicating insufficient evidence. Similarity, there were no findings of benefits in terms of reduction in the ICU LOS (standard difference in the means, −0.32 days; 95% CI, −0.71 to 0.07 days) and hospital LOS (standard difference in the means, −1.11 days; 95% CI, −2.28 to 0.06 days). Meanwhile, the results of TSA did not confirm this conclusion.

**Conclusions:**

Although conventional meta-analysis showed that early initiation of RRT in patients with AKI was not associated with decreased mortality, ICU LOS and hospital LOS, TSA indicated that the data were far too sparse to make any conclusions. Therefore, well-designed, large RCTs are needed.

## Introduction

Acute kidney injury (AKI) is a life-threatening condition in critically ill patients and has a high incidence of morbidity and mortality [1–3]. Although, in recent decades, numerous strategies, including fluid therapy, diuretic treatment, and titration of vasopressors, have been developed to reduce fatal events and improve clinical outcomes, therapies to reverse the natural course of AKI are limited, and protocol-based supportive care is still the cornerstone of treatment [4].

Renal replacement therapy (RRT) helps to remove fluid overload and waste products until the preserved kidney function is restored. Numerous cohort studies have suggested that early initiation of RRT can quickly correct internal environment disorders, such as refractory fluid overload, hyperkalemia, and severe metabolic acidosis (pH <7.1) and have associated early RRT with improved clinical outcomes [5–11]. In addition, meta-analyses based on these data also provided evidence to support early RRT in AKI [12,13].

However, the first randomized clinical trial (RCT) [14] of the early RRT, published in 2002, led to the disappointing conclusion that survival at 28 days and recovery of renal function were not improved with the use of early RRT in critically ill patients. Meanwhile, subsequent smaller RCTs also suggested that early application of RRT is deleterious in patients with septic shock [15] and cardiac surgery [16]. Therefore, it remains controversial whether early RRT can reduce the mortality of patients with AKI more than late RRT.

Two recent RCTs (AKIKI and ELAIN) reported conflicting results in relation to survival outcomes using early versus late RRT in AKI [17,18]. These trials were both rigorously designed and contributed to the largest samples to date, therefore increasing the uncertainty and controversy regarding when to initiate RRT in AKI [4,19,20]. Thus to assess the most recent available evidence, we performed a meta-analysis to compare the effect of early RRT versus late RRT in patients with AKI. We further applied trial sequential analysis (TSA) to determine whether the currently available evidence was sufficient and conclusive.

## Methods

### Search strategy and selection criteria

A systematic search of studies published between January 1, 1985, and Aug 21, 2016, was conducted using PUBMED, Cochrane Library databases, and Web of Science. Studies were identified that evaluated mortality outcomes and compared early versus late initiation of RRT in patients with AKI. The search terms used were *acute kidney injury*, *renal replacement therapy*, *time of initiation*, and *critical illness* (S1 Table). Pertinent trials were also sought at clinicaltrials.gov.

The references of original and review articles were also cross-checked. Study selection was performed by 2 of us independently (YMF and RG), with disagreements resolved by consensus among all authors. Citations were first reviewed at the title and abstract level. Full texts of all short-listed studies were then retrieved.

The present meta-analysis was performed according to the recommendations of the Cochrane Handbook for Systematic Reviews of Interventions [21] and was also done in compliance with PRISMA (Preferred Reporting Items for Systematic Reviews and Meta-Analyses statement) guidelines [22] (S2 Table). This systematic review was not registered, and a protocol does not exist. The search was limited to human subjects, and no language restrictions were applied.

The inclusion criteria were as follows: (1) study design: RCTs; (2) comparison: the effect of early versus late initiation of RRT in patients with AKI; and (3) population: critically ill adult patients (>18 years old). Exclusion criteria were as follows: (1) studies that included patients with preexisting chronic kidney disease or previous RRT; (2) data from the published results that could not be extracted and analyzed; and (3) studies that included pregnant patients.

Reference lists from the identified trials and review articles were then manually scanned to identify any additional relevant studies. The updated literature search, data extraction, and quality assessment were done independently by two authors (YMF and RG) using a standardized approach.

### Data extraction and quality assessment

Two investigators (YMF and RG) independently performed the study selection. Disagreements between the two investigators were resolved by a third party (YY). A standard form was used to collect data from each study. The form included first author, year of publication, study design, country, patient type, total number of patients, early RRT criteria and primary outcome. The primary outcome was mortality at latest follow-up, including mortality at 28, 60, and 90 days in accordance with the results from the primary authors. Secondary outcomes included intensive care unit (ICU) length of stay (LOS) and hospital LOS. Two investigators (YMF and RG) extracted the data independently.

The definition used for early initiation of RRT was consistent with the criteria used by the original authors in their respective studies. Early RRT was defined based on biochemical markers from the RIFLE classifications (risk, injury, failure, loss of function, and end-stage kidney disease), the Acute Kidney Injury Network (AKIN) stages, or time-based cutoffs (e.g., within a defined time from ICU admission or development of a biochemical “start time”).

The quality of the included studies was assessed with standard criteria: random sequence generation, allocation concealment, blinding of participants and personnel, blinding of outcome assessment, incomplete outcome data, selective reporting and other biases. When data were missing or incomplete, the original authors were contacted by written correspondence for clarification, and any relevant information obtained was included in the review.

### Grading the quality of the evidence

The quality of evidence for primary and secondary outcomes of this meta-analysis was evaluated independently by two reviewers (FZ and RG) according to the GRADE methodology for risk of bias, inconsistency, indirectness, imprecision, and publication bias; and classified as very low, low, moderate, or high. Summary tables were constructed using the GRADE Profiler (GRADEpro, version 3.6.1) [23].

### Statistical analysis

We calculated relative risks (RRs) with 95% confidence intervals (CIs) for dichotomous outcomes and mean differences with 95% CIs for continuous outcomes. Heterogeneity across studies was quantified using the *I*^*2*^ statistic; 25%≤*I*^*2*^<50% indicated low heterogeneity, 50%≤*I*^*2*^<75% indicated moderate heterogeneity, and *I*^*2*^≥75% indicated high heterogeneity. A random-effect model was used to analyze the results of trials with significant heterogeneity, and a sensitivity analysis was performed to test the robustness of results. Subgroup analysis was conducted to investigate potential sources of between-study heterogeneity. Publication bias was assessed using the Begg and Egger tests. A *P* value less than 0.05 was considered to indicate a statistically significant difference. All statistical analyses were performed using commercially available software, including Stata version 13.0 (StataCorp LP), TSA version 0.9 (Copenhagen Trial Unit) and RevMan 5.3 (Cochran IMS).

### Trial sequential analysis

Random errors are widely distributed in individual studies, such that repeated tests for significance using traditional meta-analysis may increase the risk of type I errors when sparse and accumulated data are analyzed. Therefore, it is wise to accept a tentative conclusion based on current trials, because it may be reversed by type I errors when subsequent trials are taken into account. TSA is a method that solves this important issue by adapting the monitoring boundaries to evaluate the accumulated evidence [24] and calculating the estimated information size to guide protocols in subsequent trials [25,26]. Basically, TSA recognizes a conclusion from conventional meta-analysis with 1 of the following 4 outcomes: (1) If the cumulative Z-curve exceeds the estimated information size and crosses the trial sequential monitoring boundary, the conclusion is sufficient and no more trials are needed; (2) if the cumulative Z-curve crosses the traditional monitoring boundary but does not cross the trial sequential monitoring boundary or exceed the estimated information size, the current conclusion may be a false-positive result and more trials should be included to clarify this issue; (3) if the cumulative Z-curve does not cross the traditional monitoring boundary and the trial sequential monitoring boundary, the conclusion may be a false-negative finding and more trials should be included to prove this issue; or (4) if the cumulative Z-curve exceeds the estimated information size but does not cross the traditional monitoring boundary, the negative conclusion is sufficient and there are no significant differences between the intervention group and the control group. In this study, we calculated the diversity-adjusted estimated information size using α = 0.05 (two-sided) and β = 0.20 (power 80%), with an anticipated 18% reduction in RR of mortality and an anticipated estimated –1.4 days mean difference in ICU LOS and –6.44 days estimated mean difference in hospital LOS.

## Results

### Study selection

**Fig 1:**
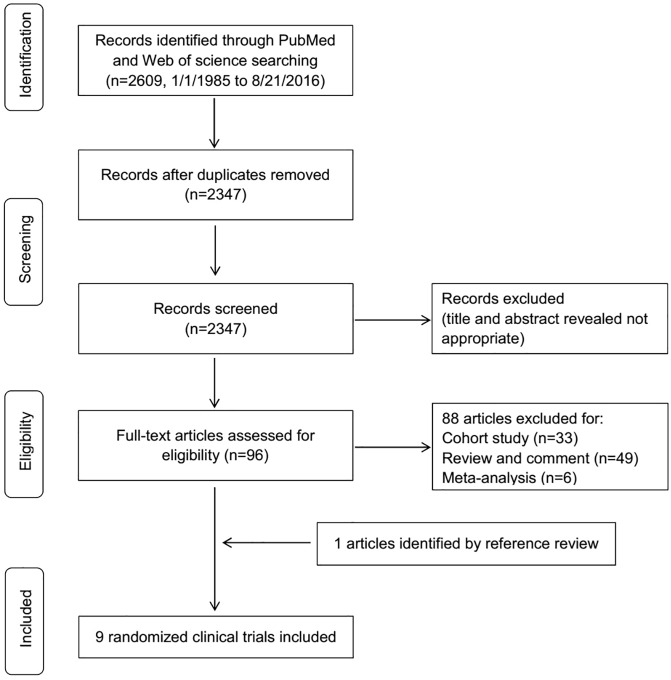
Study selection of RCTs for the meta-analysis.

The PRISMA statement flowchart shown in Fig 1 details the process of literature screening, study selection, and reasons for exclusion. The systematic search identified 2347 relevant references. After screening titles and abstracts, we excluded 2251 articles due to irrelevance to the topic or because they did not meet the inclusion criteria. Full texts of the 96 remaining articles were retrieved for formal review. After assessment of the full texts, 88 articles were excluded. After we reviewed the original articles and additional references, 9 RCTs [14–18,27–30] were included to compare the effect of early versus late RRT in patients with AKI, resulting in a total of 1636 patients in both trial arms (early RRT, n = 827; late RRT, n = 809). The two authors had no disagreements regarding study selection.

Patient characteristics and demographic data are summarized in Table 1. All trials consistently included patients with AKI who required RRT and reported mortality as the primary outcome. However, the follow-up periods varied: five trials assessed mortality within 1 month [14–16,27,29], three trials assessed mortality at more than 2 months [17,18,30], and one trial used in-hospital mortality as a benchmark [28]. Only six of the included trials reported ICU LOS and hospital LOS [14,16–18,27,30]. These trials were published between 2002 and 2016. Among these trials, four were conducted in Europe [14,15,17,18], three in Asia [27–29], and two in North America [16,30]. Five trials were multicenter studies [14–17,30]. The mean age of the patients ranged from 42 to 70 years. Severity of illness at baseline was objectively defined by eight trials using internationally recognized scoring systems [14–18,28–30].

The sample sizes in these trials varied considerably. Six trials [14,16–18,28,30] included more than 100 patients. Types of RRT also varied: three trials [14–16] applied hemofiltration, three trials [27–29] applied hemodialysis, two trials [17,30] applied a combined technique and one [18] applied hemodiafiltration. For the early RRT group, six trials [14–18,30] used time cut-off value as early criteria, and three [27–29] used biochemical criteria. Patients who were pregnant; who had chronic renal failure, received dialysis therapy before evaluation, or hepatorenal syndrome; or who were younger than 18 years old had been excluded from most of the trials.

**Table 1:** Characteristics of studies included in the meta-analysis.

Study	Year	Study Design	Country	Patient Type	RRT Type	Patients Num	Early RRT Criteria	Primary Outcome
Bouman	2002	1. RCT 2. Two-center study	Netherlands	Ventilated severely ill patients	CVVH	106	1. TIME 2. Time from randomization < 12h	1. 28 d Mortality: negative 2. EHV: 9/35(26%) died 3. ELV: 11/35(31%) died 4. LLV: 9/36(25%) died 5. p = 0.8
Durmaz	2003	1. RCT 2. Single-center study	Turkey	Cardiac surgery	CVVHD	44	1. BIOCHEM 2. Cr rise >10% from pre-op level within 48hrs of surgery	1. Hospital mortality: positive 2. Early 1/21 (4.8%) died 3. Late 7/23 (30.4%) died 4. p = 0.048
Sugahara	2004	1. RCT 2. Single-center study	Japan	Cardiac surgery	CVVHD	28	1. BIOCHEM 2. When hourly urinary output became less than 30 mL/hr for three consecutive hours (or daily urinary output was approximately 750 mL or less)	1. 14 d Mortality: positive 2. Early 2/14 (14%) died 3. Late 12/14 (86%) died 4. p<0.01
Payen	2009	1. RCT 2. Multi-center study	France	Severe sepsis or septic shock	CVVH	76	1. TIME 2. Protocolized RRT × 96hrs w/diagnosis of ‘sepsis’.	1. 14 d Mortality: negative 2. Early 20/37 (54%) died 3. Late 17/37 (44%) died 4. p = 0.49
Jamale	2010	1. RCT 2. Single-center study	India	AKI	IHD	208	1. BIOCHEM 2. When serum urea nitrogen and/or creatinine levels increased to 70 and 7 mg/dL	1. Hospital mortality: negative 2. Early 21/102 (20.5%) died 3. Late 13/106 (12%) died 4. p = 0.2
Combes	2015	1. RCT 2. Multi-center study	USA	Cardiac surgery	Mix	224	1. TIME 2. RRT initiated <24hrs and continued for min of 48hrs	1. 30 d Mortality: negative 2. Early 40/112 (36%) died 3. Late 40/112 (36%) died 4. p = 1.0
Wald	2015	1. RCT 2. Multi-center study	Canada	Critically ill patients with severe AKI	Mix	100	1. TIME 2. Time from randomization < 12h	1. 90 d Mortality: negative 2. Early 16/48 (33%) died 3. Late 19/52 (37%) died 4. p = 0.74
Gaudry	2016	1. RCT 2. Multi-center study	France	Critically ill patients with KIDGO 3 AKI	Mix	619	1. TIME 2. <6 hours after diagnosis of stage 3 AKI (KDIGO)	1. 60 d Mortality: negative 2. Early 150/311 (48%) died 3. Late 153/308 (50%) died 4. p = 0.79
Zarbock	2016	1. RCT 2. Single-center study	German	Critically ill patients with KIDGO 2 AKI	CVVHDF	231	1. TIME 2. <8 hours after diagnosis of stage 2 AKI (KDIGO)	1. 90 d Mortality: positive 2. Early 44/112 (40%) died 3. Late 65/119 (55%) died 4. p = 0.03

AKI, Acute kidey injury; KIDGO, Kidney Disease: Improving Global Outcomes; CVVH, Continuous veno-venous hemofiltration; CVVHD, Continuous veno-venous hemodialysis; IHD, intermittent haemodialysis; CVVHDF, Continuous veno-venous hemodiafiltration; EHV, Early high volume; ELV, Early low volume; LLV, Late low volume.

### Quality of studies

**Fig 2:**
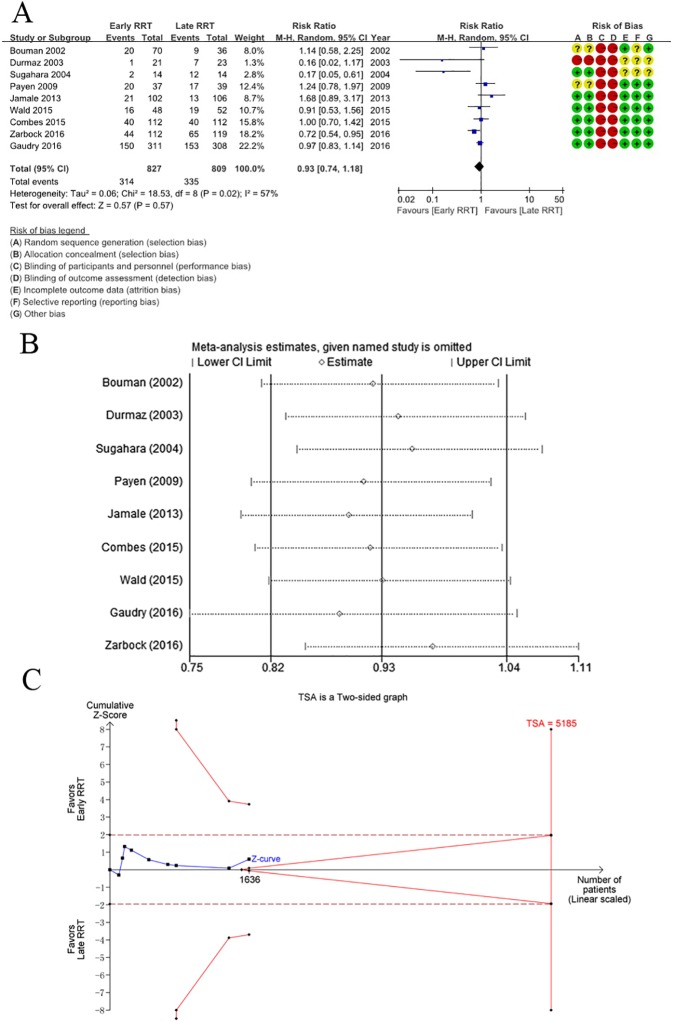
**A.** Forest plot of mortality in patients with AKI regarding early versus late initiating of RRT. Assessment for risk of bias. green = low risk of bias; yellow = uncertain risk of bias; red = high risk of bias. **B.** Sensitivity analysis of primary outcome of mortality. Single trial was excluded each time, however, pooled estimate and 95% CI had no significant changes. **C.** TSA on mortality in patients with AKI receiving early versus late initiating of RRT, which showed that the cumulative Z-curve did not cross either the conventional boundary for benefit or the trial sequential monitoring boundary for benefit. Therefore, it established insufficient and inconclusive evidence. The estimated required information size of 5185 patients was calculated using α = 0.05 (two-sided) and, β = 0.20 (power 80%), an anticipated relative risk reduction of 18%, and an event proportion of 41.4% in the late RRT group.

Details of the tool used to assess the risk of bias are shown in Fig 2A. Owing to the nature of the interventions, it was impossible for the medical staff to perform the studies blinded. Therefore, the GRADE Working Group grades of evidence were low for the primary outcome, and very low for the secondary outcomes of ICU LOS and hospital LOS. This was mainly due to the risk of bias and the moderate to high heterogeneity within studies. Full GRADE profiles for the included evidence can be found in S3 Table.

### Primary outcome

The main endpoint of mortality was defined in each individual trials, and all trials reported on patient mortality. If mortality was assessed at several time points in a study, we used data from the latest follow-up time for overall assessment of mortality. Overall mortality in these trials was 39.7% (649/1636). In the early RRT group, 38.0% (314/827) of patients died; in the late RRT group, 41.4% (335/809) of patients died. There was no significant difference in mortality between the early RRT and late RRT group (RR 0.93; 95% CI 0.74 to 1.18; *P* = 0.57), and moderate heterogeneity was found (tau^2^ = 0.06, chi^2^ = 18.53, degrees of freedom [df] = 8, *P* = 0.02, *I*^2^ = 57%; Fig 2A). To evaluate the contribution of each individual study to the moderate heterogeneity, sensitivity analysis with consecutive exclusion of one trial each time was performed. However, the meta-analyses performed after the exclusion of individual trials showed no significant effect on the pooled estimate and 95% CI (Fig 2B). Meanwhile, because of the moderate heterogeneity, the random-effect model of the DerSimonian-Laird (DL) and Sidik-Jonkman (SJ) methods was used for TSA. The results showed that the cumulative Z-curve crossed neither the traditional boundary nor the trial sequential monitoring boundary (Fig 2C), indicating insufficient power to draw a definitive conclusion.

### Secondary outcomes

**Fig 3:**
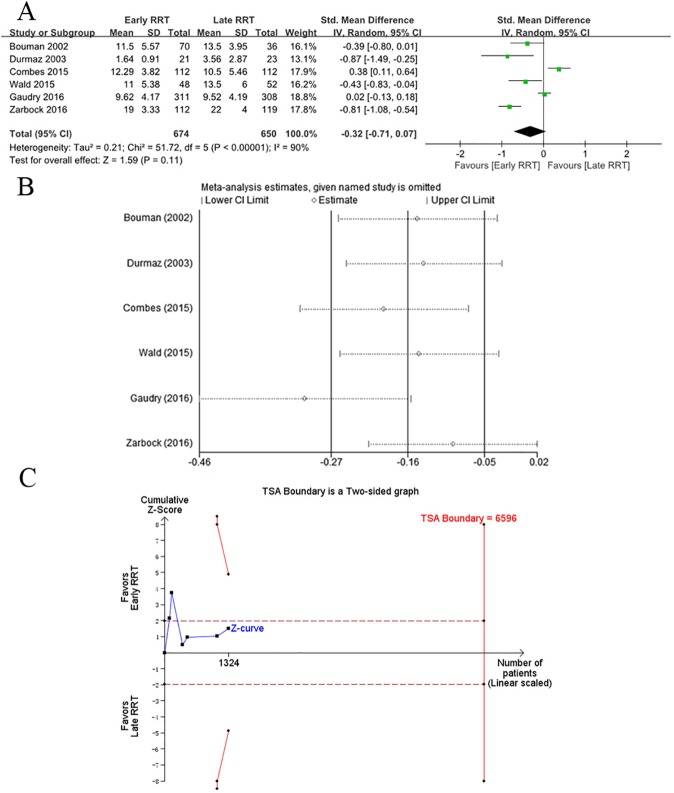
**A.** Forest plot of ICU LOS in patients with AKI regarding early versus late initiating of RRT. **B.** Sensitivity analysis of secondary outcome of ICU LOS. **C.** TSA on ICU LOS in patients with AKI receiving early versus late initiating of RRT, which showed that the cumulative Z-curve crossed the conventional boundary for benefit but did not cross the trial sequential monitoring boundary for benefit. Therefore, it established insufficient and inconclusive evidence. The estimated required information size of 6596 patients was calculated using α = 0.05 (two-sided) and β = 0.20 (power 80%), an anticipated estimated mean difference reduction of −1.4, and a heterogeneity correction of 92% in the late RRT group.

The secondary outcomes were ICU LOS and hospital LOS. Six of nine RCTs reported ICU LOS and hospital LOS [14,16–18,27,30]. Only one of these studies reported a significant decrease in ICU LOS and hospital LOS following early RRT [27]. Meta-analysis showed that there was no significant decrease in ICU LOS in patients receiving early RRT, with a standard difference in the means of −0.32 (95% CI −0.71 to 0.07, *p*<0.00001) using a random-effects model and high heterogeneity (tau^2^ = 0.21, chi^2^ = 51.72, df = 5, *p*<0.00001, *I*^2^ = 90%; Fig 3A). To evaluate the contribution of each study to the high heterogeneity, sensitivity analysis with consecutive exclusion of single trials was performed. The results of the study conducted by Gaudry et al. [17] were completely out of range of the other results and probably contributed to the heterogeneity, indicating a lack of reliability in our conclusions (Fig 3B). For TSA using α = 0.05 (two sided) and β = 80% with an estimated mean difference = −1.4, variance = 32.95, and heterogeneity correction = 92%, the Z-curve crossed the conventional boundary but did not reach the trial sequential monitoring boundary or TSA information size (Fig 3C).

**Fig 4:**
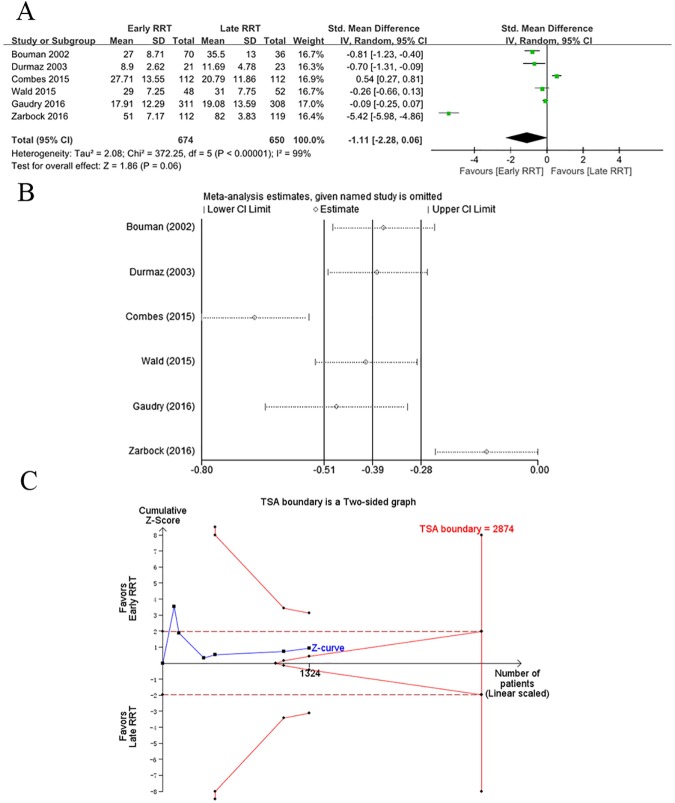
**A.** Forest plot of hospital LOS in patients with AKI regarding early versus late initiating of RRT. **B.** Sensitivity analysis of secondary outcome of hospital LOS. **C**. TSA on hospital LOS in patients with AKI receiving early versus late initiating of RRT, which showed that the cumulative Z-curve crossed the conventional boundary for benefit but did not cross the trial sequential monitoring boundary for benefit. Therefore, it established insufficient and inconclusive evidence. The estimated required information size of 2874 patients was calculated using α = 0.05 (two-sided) and β = 0.20 (power 80%), an anticipated estimated mean difference reduction of −6.44, and a heterogeneity correction of 96% in the late RRT group.

Forest plots showed that there was no significant decrease in hospital LOS in patients receiving early RRT, with a standard difference in the means of –1.11 (95% CI, −2.28 to 0.06, *p*<0.00001) using a random-effects model, as well as high heterogeneity (tau^2^ = 2.08, chi^2^ = 372.25, df = 5, *I*^2^ = 99%; Fig 4A). To evaluate the contribution of individual studies to the high heterogeneity, sensitivity analysis with consecutive exclusion of individual trials was performed. The results of the studies conducted by Combes et al [16] and Zarbock et al [18] were completely out of range of the other results and probably contributed to the heterogeneity, indicating a lack of reliability in our conclusions (Fig 4B). For TSA using α = 0.05 (two-sided) and β = 80% with estimated mean difference = −6.44, variance = 151.87, and heterogeneity correction = 96%, the Z-curve crossed the conventional boundary but did not reach the trial sequential monitoring boundary and TSA information size (Fig 4C).

### Publication bias

We assessed potential publication bias for the primary outcome of mortality (*P* = 0.52 for the Begg test, *P* = 0.473 for the Egger test) and the secondary outcomes of ICU LOS (*P* = 0.75 for the Begg test, *P* = 0.063 for the Egger test) and hospital LOS (*P* = 0.754 for the Begg test, *P* = 0.547 for the Egger test). No potential publication bias was observed among the included trials (see S1 Fig).

## Discussion

RRT represents a cornerstone in the management of life-threatening AKI. Several aspects of RRT are now well established, but others remain controversial, especially the optimal timing to initiate RRT. To date, there has been clear consensus that timely RRT is required in life-threatening conditions, such as severe hyperkalemia, marked metabolic acidosis, and/or fluid overload; however, in most critically ill patients with AKI, the decision to initiate RRT is not done mainly on account of overtly life-threatening conditions [31]. Consequently, there are large variations in the timing of RRT initiation in these populations, influencing clinical outcomes. Our meta-analysis, based on the traditional method, provided two conclusions. First, early RRT was not associated with improved clinical outcomes (i.e., reductions in mortality), although this finding should be interpreted with caution, because TSA suggested this evidence may represent a false-negative result. Second, meanwhile, there were no findings to support the idea that early RRT will shorten the ICU LOS and hospital LOS. TSA indicated that the evidence was insufficient and inconclusive.

### Relation to other reviews and implication for future research

Because high-quality RCTs are limited, much of the current evidence is derived from observational studies or meta-analyses [12,13,32]. Liu [33] included 9 retrospective cohort studies and 2 RCTs (841 patients). This meta-analysis showed that early initiation of RRT in patients with AKI after cardiac surgery resulted in lower mortality at 28 days (OR = 0.29, 95% CI, 0.16–0.52, *P*<0.0001, *I*^2^ = 56%) and shorter ICU LOS (3.9 [1.5–6.3] days, *P*<0.0001, *I*^2^ = 99%). Although the majority of these clinical data appear to support the view that early RRT reduces mortality in patients with AKI, these observational data may suffer from a number of potential methodological limitations, leading to biased results [33].

In addition, our meta-analysis included 9 RCTs with a total of 1636 participants. Hence, we had more sample size as well as updated trials and a higher level of evidence coming from RCTs. Thus, evidence based on our conclusions should have more strength to demonstrate this issue. More importantly, we further performed TSA to assess the robustness of these outcomes which indicated that there was no conclusive evidence to support or oppose the early initiation of RRT in patients with AKI. It is, therefore, encouraging high-quality RCTs to find the truth.

Wierstra et al [34] synthesized the largest pool of clinical data to date (7 RCTs, 10 prospective cohorts, and 19 retrospective cohorts) and concluded that early initiation of RRT in critical illness complicated by AKI did not improve patient survival or reduce ICU or hospital LOS. Although our findings also did not observe significant benefits associated with early initiation of RRT in patients with AKI, our conclusions were based on RCTs only. Because observational studies make no intervention and patients are allocated treatment based on clinical decisions, selection bias may exist, thereby overestimating the benefits of early RRT. Our evidence minimizes this source of bias and may thus be considered more reliable.

In addition, we included 2 more recently published high-quality clinical trials (the ELAIN and AKIKI trials) than Wierstra et al [34]. Although the number of RCTs increased by only 2 clinical trials, the total number of patients involved doubled, from 786 to 1636. Therefore, our analysis may provide stronger conclusions on this issue.

Although a recent meta-analysis of RCTs [35] included the ELAIN and AKIKI trials (6 RCTs with 1257 patients) and made conclusions similar to ours, their search strategy was incomplete. Also, our meta-analysis included 3 additional trials, for a total of 1636 participants (9 RCTs). Furthermore, because the available RCTs had small sample sizes (only 1 trial [17] included more than 300 patients) and the number of RCTs was insufficient (fewer than 10 RCTs), there was no additional ability to determine the risk of random errors due to repeated testing of the accumulated data in the current conclusive meta-analysis. To our knowledge, this is the first study to investigate this effect using TSA method. Although our meta-analysis involved more data and thus should have conclusions with greater power, TSA showed that the optimal timing of initiating RRT remains unclear. More RCTs should be performed to further clarify this issue.

Fortunately, there are two ongoing RCTs that should provide additional information regarding the optimal timing of RRT in critically ill patients with AKI, with special interest in patients with septic shock (IDEAL-ICU) or admitted to general ICU (STARRT-AKI). As well, these clinical trials have enrolled more patients. In addition to short-term follow-up, IDEAL-ICU further investigates the impact of the two RRT strategies on mortality at 180 and 360 days. Meanwhile, more diverse outcomes are being measured, including cost-effectiveness and new inclusion criteria. Accordingly, these efforts may give physicians better approaches to real-world RRT in patients with AKI.

On the other hand, another implication from our meta-analysis should be noted for planned and ongoing clinical trials. Because there were large variations in the definition of “early” and “late” RRT among physicians and countries (for example, the “late” group in the ELAIN trial was actually earlier than the “early” group in AKIKI), moderate heterogeneity regarding the possible association between timing and mortality was found in our meta analysis. Most trials included in this analysis [14–18,30] used a fixed time point for the timing of RRT initiation with a given anticipated course of AKI. However, the precise time of AKI is elusive and a variety anticipated course of AKI. However, the precise timing of AKI is elusive. A variety of surrogate biomarkers have been used to better define this issue; neutrophil gelatinase-associated lipocalin is the most frequently investigated, and the data are promising [36,37]. However, it is important to remember that a clinical decision to start RRT is inappropriate when it does not take into account a patient’s individual condition, specific course of illness, and the different RRT modalities and dosage options that are available.

### Strengths and limitations

A major strength of this meta-analysis is the compliance with the PRISMA guidelines and the recommendations of the Cochrane Collaboration, although the protocol of our study was not registered in the international prospective register of systematic reviews (PROSPERO). To increase the robustness of our meta-analysis, we applied TSA to assess the impact of random error and repetitive testing. Finally, we evaluated the quality of evidence for the outcomes using GRADE to help healthcare professionals make better clinical decisions.

Our meta-analysis also has some limitations. The included trials in our meta-analysis were conducted on varying numbers and types of patients, had different designs, and used different criteria to determine early versus latte RRT, the method of RRT treatment, and duration of mortality follow-up. Thus, the risk of introducing potentially significant heterogeneity is imminent. In addition, double-blinding was not performed because of the features of the trials, which may have contributed to performance and detection bias.

## Conclusions

Conventional meta-analyses that included recent trial data showed that early initiation of RRT in patients with AKI was not associated with decreased mortality, ICU LOS, or hospital LOS. After TSA adjustment for sparse data and multiple update in the cumulative meta-analysis, we were unable to draw definitive conclusions regarding the ideal timing of RRT in patients with AKI. The results of ongoing and future well-designed, large RCTs are needed to clarify this issue.

## Supporting information

S1 Fig **A.** Publication bias for the primary outcome of mortality using Begg’s test. **B.** Publication bias for the primary outcome of mortality using Egger’s test. **C.** Publication bias for the secondary outcome of ICU LOS using Begg’s test. **D.** Publication bias for the secondary outcome of ICU LOS using Egger’s test. **E.** Publication bias for the secondary outcome of hospital LOS using Begg’s test. **F.** Publication bias for the secondary outcome of hospital LOS using Egger’s test.
(TIF)

S1 Table Full electronic search strategy. (DOCX)

S2 Table The PRISMA checklist. (DOC)

S3 Table The GRADE Evidence Profile for the Primary and Secondary outcomes of this meta-analysis. GRADE Working Group grades of evidence High quality: Further research is very unlikely to change our confidence in the estimate of effect. Moderate quality: Further research is likely to have an important impact on our confidence in the estimate of effect and may change the estimate. Low quality: Further research is very likely to have an important impact on our confidence in the estimate of effect and is likely to change the estimate. Very low quality: We are very uncertain about the estimate. ^1^ Although most of included RCTs were judged as high risk of performance bias because of without blinding of participants and personnel, the predefined objective outcome was just partly influenced. ^2^ Heterogeneity (*I*^2^ = 57%) as found. ^3^ Heterogeneity (*I*^2^ = 90%) was found. ^4^ Heterogeneity (*I*^2^ = 99%) was found.
(TIF)

## References

[1] Mehta RL, Burdmann EA, Cerda J, Feehally J, Finkelstein F, Garcia-Garcia G, et al (2016) Recognition and management of acute kidney injury in the International Society of Nephrology 0by25 Global Snapshot: a multinational cross-sectional study. Lancet 387: 2017–2025. 10.1016/S0140-6736(16)30240-9 27086173

[2] Gallagher M, Cass A, Bellomo R, Finfer S, Gattas D, Lee J, et al (2014) Long-term survival and dialysis dependency following acute kidney injury in intensive care: extended follow-up of a randomized controlled trial. PLoS Med 11: e1001601 10.1371/journal.pmed.1001601 24523666 PMC3921111

[3] Bihorac A, Schold JD, Hobson CE (2010) Long-term mortality associated with acute kidney injury requiring dialysis. JAMA 303: 229; author reply 229–230. 10.1001/jama.2009.1878 20085947

[4] Bagshaw SM, Wald R (2016) Acute kidney injury: Timing of renal replacement therapy in AKI. Nat Rev Nephrol. 10.1038/nrneph.2016.92 27345244

[5] Boussekey N, Capron B, Delannoy PY, Devos P, Alfandari S, Chiche A, et al (2012) Survival in critically ill patients with acute kidney injury treated with early hemodiafiltration. Int J Artif Organs 35: 1039–1046. 10.5301/ijao.5000133 23065871

[6] Carl DE, Grossman C, Behnke M, Sessler CN, Gehr TWB (2010) Effect of timing of dialysis on mortality in critically ill, septic patients with acute renal failure. Hemodialysis International 14: 11–17. 10.1111/j.1542-4758.2009.00407.x 20377649

[7] Gettings LG, Reynolds HN, Scalea T (1999) Outcome in post-traumatic acute renal failure when continuous renal replacement therapy is applied early vs. late. Intensive Care Med 25: 805–813. 10447537 10.1007/s001340050956

[8] Iyem H, Tavli M, Akcicek F, Buket S (2009) Importance of early dialysis for acute renal failure after an open-heart surgery. Hemodial Int 13: 55–61. 10.1111/j.1542-4758.2009.00347.x 19210279

[9] Ji Q, Mei Y, Wang X, Feng J, Cai J, Zhou Y, et al (2011) Timing of continuous veno-venous hemodialysis in the treatment of acute renal failure following cardiac surgery. Heart and Vessels 26: 183–189. 10.1007/s00380-010-0045-9 21063880

[10] Shiao CC, Wu VC, Li WY, Lin YF, Hu FC, Young GH, et al (2009) Late initiation of renal replacement therapy is associated with worse outcomes in acute kidney injury after major abdominal surgery. Crit Care 13: R171 10.1186/cc8147 19878554 PMC2784403

[11] Vats HS, Dart RA, Okon TR, Liang H, Paganini EP (2011) Does Early Initiation of Continuous Renal Replacement Therapy Affect Outcome: Experience in a Tertiary Care Center. Renal Failure 33: 698–706. 10.3109/0886022X.2011.589945 21787161

[12] Karvellas CJ, Farhat MR, Sajjad I, Mogensen SS, Leung AA, Wald R, et al (2011) A comparison of early versus late initiation of renal replacement therapy in critically ill patients with acute kidney injury: a systematic review and meta-analysis. Crit Care 15: R72 10.1186/cc10061 21352532 PMC3222005

[13] Shiao CC, Ko WJ, Wu VC, Huang TM, Lai CF, Lin YF, et al (2012) U-curve association between timing of renal replacement therapy initiation and in-hospital mortality in postoperative acute kidney injury. PLoS One 7: e42952 10.1371/journal.pone.0042952 22952623 PMC3429468

[14] Bouman CS, Oudemans-Van Straaten HM, Tijssen JG, Zandstra DF, Kesecioglu J (2002) Effects of early high-volume continuous venovenous hemofiltration on survival and recovery of renal function in intensive care patients with acute renal failure: a prospective, randomized trial. Crit Care Med 30: 2205–2211. 10.1097/01.CCM.0000030444.21921.EF 12394945

[15] Payen D, Mateo J, Cavaillon JM, Fraisse F, Floriot C, Vicaut E, et al (2009) Impact of continuous venovenous hemofiltration on organ failure during the early phase of severe sepsis: a randomized controlled trial. Crit Care Med 37: 803–810. 10.1097/CCM.0b013e3181962316 19237881

[16] Combes A, Brechot N, Amour J, Cozic N, Lebreton G, Guidon C, et al (2015) Early High-Volume Hemofiltration versus Standard Care for Post-Cardiac Surgery Shock. The HEROICS Study. Am J Respir Crit Care Med 192: 1179–1190. 10.1164/rccm.201503-0516OC 26167637

[17] Gaudry S, Hajage D, Schortgen F, Martin-Lefevre L, Pons B, Boulet E, et al (2016) Initiation Strategies for Renal-Replacement Therapy in the Intensive Care Unit. N Engl J Med 375: 122–133. 10.1056/NEJMoa1603017 27181456

[18] Zarbock A, Kellum JA, Schmidt C, Van Aken H, Wempe C, Pavenstadt H, et al (2016) Effect of Early vs Delayed Initiation of Renal Replacement Therapy on Mortality in Critically Ill Patients With Acute Kidney Injury: The ELAIN Randomized Clinical Trial. JAMA 315: 2190–2199. 10.1001/jama.2016.5828 27209269

[19] Chertow GM, Winkelmayer WC (2016) Early to Dialyze: Healthy and Wise? JAMA 315: 2171–2172. 10.1001/jama.2016.6210 27209075

[20] Mehta RL (2016) Renal-Replacement Therapy in the Critically Ill—Does Timing Matter? N Engl J Med 375: 175–176. 10.1056/NEJMe1606125 27181293

[21] Higgins JPT, Green S (2011) Cochrane Handbook for Systematic Reviews of Interventions Version 5.1.0; Higgins JPT, Green S, editors: John Wiley & Sons, Ltd i–xxi p.

[22] Moher D, Liberati A, Tetzlaff J, Altman DG, Group P (2009) Preferred reporting items for systematic reviews and meta-analyses: the PRISMA statement. BMJ 339: b2535 10.1136/bmj.b2535 19622551 PMC2714657

[23] Guyatt GH, Oxman AD, Vist GE, Kunz R, Falck-Ytter Y, Alonso-Coello P, et al (2008) GRADE: an emerging consensus on rating quality of evidence and strength of recommendations. BMJ 336: 924–926. 10.1136/bmj.39489.470347.AD 18436948 PMC2335261

[24] Wetterslev J, Thorlund K, Brok J, Gluud C (2008) Trial sequential analysis may establish when firm evidence is reached in cumulative meta-analysis. J Clin Epidemiol 61: 64–75. 10.1016/j.jclinepi.2007.03.013 18083463

[25] Thorlund K, Devereaux PJ, Wetterslev J, Guyatt G, Ioannidis JP, Thabane L, et al (2009) Can trial sequential monitoring boundaries reduce spurious inferences from meta-analyses? Int J Epidemiol 38: 276–286. 10.1093/ije/dyn179 18824467

[26] Wetterslev J, Thorlund K, Brok J, Gluud C (2009) Estimating required information size by quantifying diversity in random-effects model meta-analyses. BMC Med Res Methodol 9: 86 10.1186/1471-2288-9-86 20042080 PMC2809074

[27] Durmaz I, Yagdi T, Calkavur T, Mahmudov R, Apaydin AZ, Posacioglu H, et al (2003) Prophylactic dialysis in patients with renal dysfunction undergoing on-pump coronary artery bypass surgery. Ann Thorac Surg 75: 859–864. 12645707 10.1016/s0003-4975(02)04635-0

[28] Jamale TE, Hase NK, Kulkarni M, Pradeep KJ, Keskar V, Jawale S, et al (2013) Earlier-start versus usual-start dialysis in patients with community-acquired acute kidney injury: a randomized controlled trial. Am J Kidney Dis 62: 1116–1121. 10.1053/j.ajkd.2013.06.012 23932821

[29] Sugahara S, Suzuki H (2004) Early start on continuous hemodialysis therapy improves survival rate in patients with acute renal failure following coronary bypass surgery. Hemodial Int 8: 320–325. 10.1111/j.1492-7535.2004.80404.x 19379436

[30] Wald R, Adhikari NK, Smith OM, Weir MA, Pope K, Cohen A, et al (2015) Comparison of standard and accelerated initiation of renal replacement therapy in acute kidney injury. Kidney Int 88: 897–904. 10.1038/ki.2015.184 26154928

[31] Ostermann M, Wald R, Bagshaw SM (2016) Timing of Renal Replacement Therapy in Acute Kidney Injury. Contrib Nephrol 187: 106–120. 10.1159/000442369 26882338

[32] Seabra VF, Balk EM, Liangos O, Sosa MA, Cendoroglo M, Jaber BL (2008) Timing of renal replacement therapy initiation in acute renal failure: a meta-analysis. Am J Kidney Dis 52: 272–284. 10.1053/j.ajkd.2008.02.371 18562058

[33] Liu Y, Davari-Farid S, Arora P, Porhomayon J, Nader ND (2014) Early versus late initiation of renal replacement therapy in critically ill patients with acute kidney injury after cardiac surgery: a systematic review and meta-analysis. J Cardiothorac Vasc Anesth 28: 557–563. 10.1053/j.jvca.2013.12.030 24731742

[34] Wierstra BT, Kadri S, Alomar S, Burbano X, Barrisford GW, Kao RL (2016) The impact of "early" versus "late" initiation of renal replacement therapy in critical care patients with acute kidney injury: a systematic review and evidence synthesis. Crit Care 20: 122 10.1186/s13054-016-1291-8 27149861 PMC4858821

[35] Xu Y, Gao J, Zheng X, Zhong B, Na Y, Wei J (2016) Timing of initiation of renal replacement therapy for acute kidney injury: a systematic review and meta-analysis of randomized-controlled trials. Clin Exp Nephrol. 10.1007/s10157-016-1316-2 27485542

[36] Cruz DN, de Cal M, Garzotto F, Perazella MA, Lentini P, Corradi V, et al (2010) Plasma neutrophil gelatinase-associated lipocalin is an early biomarker for acute kidney injury in an adult ICU population. Intensive Care Med 36: 444–451. 10.1007/s00134-009-1711-1 19956925 PMC2820221

[37] Mishra J, Dent C, Tarabishi R, Mitsnefes MM, Ma Q, Kelly C, et al (2005) Neutrophil gelatinase-associated lipocalin (NGAL) as a biomarker for acute renal injury after cardiac surgery. Lancet 365: 1231–1238. 10.1016/S0140-6736(05)74811-X 15811456

