# Supplementary figures and images for: The effect of early versus late initiation of renal replacement therapy in patients with acute kidney injury: A meta-analysis with trial sequential analysis of randomized controlled trials
Source: PLoS One. 2017 Mar 22;12(3):e0174158. doi: 10.1371/journal.pone.0174158 (PMC5362192; doi:10.1371/journal.pone.0174158)

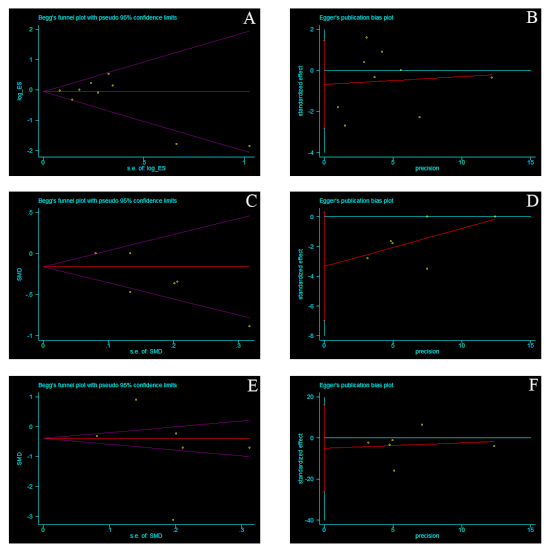

Supplement: S1 Fig — A. Publication bias for the primary outcome of mortality using Begg’s test. B. Publication bias for the primary outcome of mortality using Egger’s test. C. Publication bias for the secondary outcome of ICU LOS using Begg’s test. D. Publication bias for the secondary outcome of ICU LOS using Egger’s test. E. Publication bias for the secondary outcome of hospital LOS using Begg’s test. F. Publication bias for the secondary outcome of hospital LOS using Egger’s test. (TIF) [file pone.0174158.s001.tif]

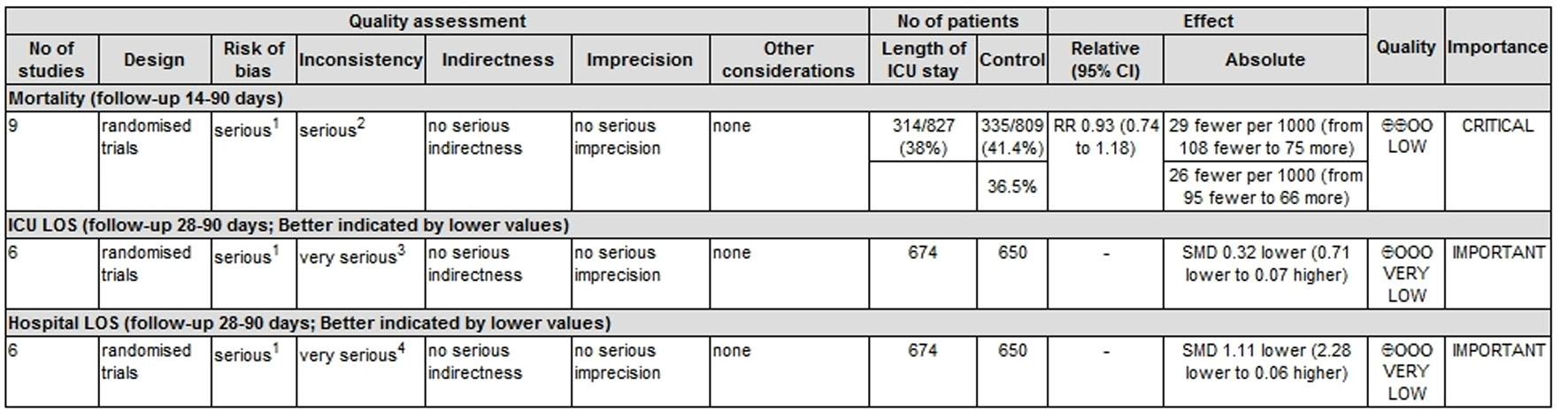

Supplement: S3 Table — GRADE Working Group grades of evidence High quality: Further research is very unlikely to change our confidence in the estimate of effect. Moderate quality: Further research is likely to have an important impact on our confidence in the estimate of effect and may change the estimate. Low quality: Further research is very likely to have an important impact on our confidence in the estimate of effect and is likely to change the estimate. Very low quality: We are very uncertain about the estimate. 1 Although most of included RCTs were judged as high risk of performance bias because of without blinding of participants and personnel, the predefined objective outcome was just partly influenced. 2 Heterogeneity (I2 = 57%) as found. 3 Heterogeneity (I2 = 90%) was found. 4 Heterogeneity (I2 = 99%) was found. (TIF) [file pone.0174158.s004.tif]
