# Supplementary material for: The effect of early versus late initiation of renal replacement therapy in patients with acute kidney injury: A meta-analysis with trial sequential analysis of randomized controlled trials
Source: PLoS One. 2017 Mar 22;12(3):e0174158. doi: 10.1371/journal.pone.0174158 (PMC5362192; doi:10.1371/journal.pone.0174158)
Supplement: S1 Table — (DOCX) [file pone.0174158.s002.docx]

| PUBMED, searched 31 Jun 2016 | | |
| --- | --- | --- |
| 1 | Acute kidney injury [MeSH majr] OR “acute kidney”[ti] OR “acute renal”[ti] | 52,709 |
| 2 | Renal replacement therapy [MeSH majr] OR dialysis [ti] OR dialyzed [ti] OR dialyzing [ti] OR hemodialysis [ti] OR hemofiltration [ti] | 226,383 |
| 3 | Time to treatment [MeSH] OR Time factors [MeSH] OR Early [ti/ab] OR earlier [ti/ab] OR time [ti/ab] OR timing [ti/ab] OR accelerate [ti/ab] OR accelerated [ti/ab] OR accelerating [ti/ab] OR acceleration [ti/ab] OR late [ti/ab] OR Delayed [ti/ab] | 4,542,586 |
| 4 | Combine #1 AND # 2 AND # 3 | 4246 |
| 5 | Filters:  Randomized controlled trial[Publication Type] OR randomized [Title/Abstract] OR placebo [Title/Abstract] | 647,599 |
| 6 | Filters: English | 21,561,071 |
| 7 | Combine #4 AND # 5 AND # 6 | 307 |
| 8 | Removal of duplicates (1) | 1,003 |

| EMBASE, searched 31 Jun 2016 | | |
| --- | --- | --- |
| 1 | Acute kidney failure OR Acute kidney tubule necrosis OR acute kidney OR acute renal | 69,225 |
| 2 | Renal replacement therapy OR dialysis OR dialyzed OR dialyzing OR hemodialysis OR hemofiltration | 221,772 |
| 3 | Time to treatment OR Time OR Early intervention OR Early OR Earlier OR Timing OR Accelerated OR Accelerating OR Acceleration OR Late | 5,026,658 |
| 4 | Critical Care OR Intensive Care Unit OR ICU | 306,234 |
| 5 | Combine #1 AND # 2 AND # 3 AND #2 | 1,897 |
| 6 | Limit to: English | 1,780 |
| 7 | Removal of duplicates (378) | 1,402 |
